# Phytochemical Characterization and Bioactive Potential of *Stachys germanica* L.: LC–MS/MS Profiling and Multi‐Target Biological Activities

**DOI:** 10.1002/fsn3.72238

**Published:** 2026-08-07

**Authors:** Gözde Gülin İnan, Derya Doğanay, Yiğit İnan, Selin Akyüz‐Çetin, Neşet Neşetoğlu, İbrahim Daniş, Esra Mertoğlu, Durişehvar Özer Ünal, Gokhan Zengin

**Affiliations:** ^1^ Department of Analytical Chemistry, Faculty of Pharmacy University of Health Sciences İstanbul Türkiye; ^2^ Department of Pharmaceutical Microbiology, Faculty of Pharmacy University of Health Sciences İstanbul Türkiye; ^3^ Department of Pharmacognosy, Faculty of Pharmacy Kent University İstanbul Türkiye; ^4^ Department of Pharmacognosy, Faculty of Pharmacy Bahcesehir University İstanbul Türkiye; ^5^ Department of Analytical Chemistry, Faculty of Pharmacy Istanbul University İstanbul Türkiye; ^6^ Drug Application and Research Center Istanbul University Istanbul Turkey; ^7^ Department of Phytotherapy, Hamidiye Institute of Health Sciences University of Health Sciences Istanbul Turkey; ^8^ Department of Biology, Science Faculty Selcuk University Konya Turkey

**Keywords:** antimicrobial potential, antioxidant activity, enzyme inhibition, phenolic compounds, Stachys germanica

## Abstract

*Stachys germanica* L. is a member of the Lamiaceae family traditionally recognized for its bioactive secondary metabolites. The present study aimed to investigate the phytochemical composition and biological potential of the 80% ethanolic extract of *S. germanica* using spectrophotometric assays and liquid chromatography–tandem mass spectrometry (LC–MS/MS) analysis. The extract exhibited a high phenolic content (59.80 ± 0.84 mg GAE/g) and flavonoid content (20.50 ± 0.24 mg RE/g), with notable antioxidant activity, particularly in CUPRAC (445.26 ± 6.11 mg TE/g), ABTS (387.12 ± 3.03 mg TE/g), FRAP (360.89 ± 5.19 mg TE/g), and DPPH (333.96 ± 1.61 mg TE/g) assays. Enzyme inhibition assays revealed a selective inhibitory profile, with pronounced activity against tyrosinase (62.23 ± 0.06 mg KAE/g) and α‐glucosidase (1.05 ± 0.24 mmol ACAE/g), suggesting potential relevance in dermatological and metabolic applications. Notably, antimicrobial evaluation demonstrated significant activity against *Acinetobacter baumannii* ATCC 19606 (MIC: 97 μg/mL), highlighting a selective antibacterial effect against a clinically relevant Gram‐negative pathogen. LC–MS/MS analysis revealed a chemically diverse metabolite profile largely composed of phenolic acids and flavonoids, with chlorogenic acid (1024 ± 20.54 μg/g), naringin (196.10 ± 4.65 μg/g), luteolin (82.46 ± 1.88 μg/g), and kaempferol (70.99 ± 1.71 μg/g) identified as the main components. Overall, this study provides a multi‐dimensional characterization of *S. germanica* , linking its phenolic‐rich composition to antioxidant, enzyme‐modulating, and selective antimicrobial activities. These findings highlight the potential of *S. germanica* as a natural source of bioactive compounds for food, nutraceutical, and functional product applications.

## Graphical abstract

The 80% ethanolic extract of *Stachys germanica* L. exhibited a phenolic‐rich phytochemical profile characterized by chlorogenic acid, naringin, luteolin, and kaempferol. The extract demonstrated notable antioxidant capacity, selective tyrosinase and α‐glucosidase inhibition, and significant antibacterial activity against *Acinetobacter baumannii* ATCC 19606, supporting its potential for functional food, nutraceutical, and cosmeceutical applications.

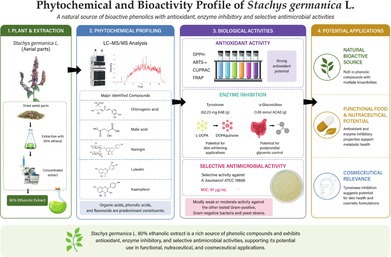

## 1 Introduction

The genus *Stachys* L., belonging to the Lamiaceae family, is one of the most extensive taxonomic groups within the family, encompassing approximately 300 species distributed across various ecological zones worldwide (Tundis et al. 2014). Turkey is documented as a significant center of diversity for this genus, harboring over 90 species with a remarkable endemism rate of approximately 47% (Akçiçek et al. 2012). Members of this genus are typically distinguished by their densely villous (hairy) leaves and distinct verticillate inflorescences. They are known colloquially as ‘mountain tea’ (dağ çayı) or ‘woundwort’ (Satıl and Açar 2020).

In the realm of ethnobotany, *Stachys* species have been utilized for centuries and continue to attract scientific attention due to their broad therapeutic relevance (Ivanova et al. 2023). Accordingly, ethnomedicinal applications of *Stachys* taxa have been reported across Europe, the Mediterranean basin, Central Asia, and the Middle East for the treatment of inflammatory disorders, gastrointestinal complaints, respiratory conditions, wound healing, and infectious diseases (Mamedov et al. 2005; Mantovska et al. 2022). In Anatolian folk medicine, *Stachys* species have been traditionally used both internally and externally, where decoctions or aqueous extracts are consumed for digestive disorders, anti‐diabetic purposes, inflammatory conditions, and general metabolic support, while topical preparations such as poultices and compresses are applied for wound healing, skin infections, burns, and inflammatory skin disorders (Gören 2014). More specifically, in Iranian folk medicine, the flowers of *S. germanica* have been reported to be used as an infusion for gastrodynia and painful menstruation (Naghibi et al. 2005).

The genus is characterized by a remarkable diversity of secondary metabolites, predominantly comprising phenylethanoid glycosides (verbascoside, isoverbascoside), iridoids (harpagide, acetylharpagide), flavonoids (apigenin, luteolin, and their derivatives), phenolic acids (chlorogenic, caffeic, and rosmarinic acids), and diterpenes (Napolitano et al. 2022). These bioactive constituents are responsible for a broad spectrum of pharmacological actions, including antioxidant, antimicrobial, anti‐inflammatory, and cytotoxic activities (Conforti et al. 2009). Among these biological effects, antioxidant activity is of particular relevance, as oxidative stress, arising from an imbalance between the production of reactive oxygen species and the endogenous antioxidant defense system, is a critical factor in the pathogenesis of various chronic conditions, such as cardiovascular diseases, neurodegeneration, and cancer (İnan et al. 2021). In this context, the therapeutic potential of *Stachys* species extends to the modulation of key metabolic pathways. In particular, the inhibition of cholinesterases (AChE and BChE), which are central to the progression of Alzheimer's disease, as well as carbohydrate‐hydrolyzing enzymes (α‐amylase and α‐glucosidase), which serve as primary targets in the management of type 2 diabetes, has been extensively documented in recent studies (Zengin et al. 2016).

Accordingly, the present study focuses on conducting a comprehensive phytochemical characterization of *S. germanica* extract using LC–MS/MS and evaluating its antioxidant, enzyme‐inhibitory, and antimicrobial activities. To the best of our knowledge, this is one of the first studies to integrate LC–MS/MS‐based quantitative metabolite profiling with antioxidant, enzyme‐inhibitory, and antimicrobial evaluations for *S. germanica* . By linking the identified chemical composition to biological responses, this research aims to elucidate the species' multifunctional bioactive potential, particularly its role in modulating oxidative stress and metabolic enzymes. The findings from this study provide a scientific basis for the potential use of *S. germanica* in pharmaceutical, nutraceutical, and cosmeceutical applications, while also contributing to current knowledge of its chemical diversity and therapeutic value. In recent years, plant‐derived phenolics have gained increasing attention in food science due to their potential as natural antioxidants, preservatives, and functional ingredients. In this context, exploring underutilized species such as *Stachys germanica* may provide valuable insights for the development of novel functional food formulations.

## 2 Materials and Methods

### 2.1 Plant Samples

The aerial parts of *S. germanica* were collected in May 2023 from the vicinity of Emiryakup Neighborhood, Hayrabolu district, Tekirdağ Province, Turkey. The taxonomic identification of the collected plant material was carried out by Prof. Dr. Galip Akaydın. A voucher specimen was deposited in the herbarium of Hacettepe University, Faculty of Education, Department of Mathematics and Science Education, Biology Education Division, with the voucher number HEF17228.

### 2.2 Chemicals and Reagents

Analytical standards, dimethyl sulfoxide (DMSO), formic acid, ammonium formate (5 mM), and LC/MS‐ or HPLC‐grade organic solvents, including methanol and ethanol, were supplied by Sigma‐Aldrich (St. Louis, MO, USA). Ultrapure water used throughout the analyses was produced using a Milli‐Q Integral 3 purification system (Millipore, Billerica, MA, USA).

### 2.3 Preparation of Extract

Powdered aerial parts of *S. germanica* (200 g) were extracted with 2 L of 80% ethanol (v/v) by maceration at room temperature for 48 h. The extractive solution was separated from the plant residue by filtration and concentrated under reduced pressure using a rotary evaporator. The concentrated extract was subsequently lyophilized to obtain a dry extract powder. The final dried extract weighed 22.46 g, corresponding to an extraction yield of 11.23%. The dry extract was kept at 4°C until use in the bioactivity assays.

### 2.4 Microorganisms

The antimicrobial assays were carried out against a panel comprising both ATCC reference strains and hospital‐acquired clinical isolates. The reference strains included *Staphylococcus aureus* ATCC 29213, *Staphylococcus epidermidis* ATCC 12228, *Bacillus subtilis* ATCC 6633, *Escherichia coli* ATCC 25922, *Salmonella enterica* ATCC 13076, *Acinetobacter baumannii* ATCC 19606, *Pseudomonas aeruginosa* ATCC 27853, *Candida albicans* ATCC 10231, *Candida tropicalis* ATCC 750, and *Candida parapsilosis* ATCC 22019. The clinical isolates consisted of *Corynebacterium striatum* , *Staphylococcus aureus* , *Staphylococcus epidermidis* , *Escherichia coli* , *Pseudomonas aeruginosa* , *Acinetobacter baumannii* , and *Candida albicans* . Before testing, fresh cultures were prepared by subculturing bacterial strains on tryptic soy agar (TSA) and yeast strains on Sabouraud dextrose agar (SDA). Bacterial cultures were incubated at 37°C, whereas yeasts were incubated at 27°C for 24–48 h. The microbial suspensions were then adjusted to the turbidity of a 0.5 McFarland standard (10^6^ CFU/mL) in Mueller–Hinton broth (MHB) using a digital McFarland densitometer (BioSan), following Clinical and Laboratory Standards Institute (CLSI) recommendations (Clinical and Laboratory Standards Institute (CLSI) 2012; Clinical and Laboratory Standards Institute (CLSI) 2023).

### 2.5 Determination of Minimum Inhibitory Concentration (MIC)

The antimicrobial activity of the extract was determined by the broth microdilution assay following Clinical and Laboratory Standards Institute (CLSI) recommendations (Clinical and Laboratory Standards Institute (CLSI) 2023), with minor adaptation from the procedure described by Beargie et al. (1965). Mueller–Hinton broth (MHB) was used for bacterial strains, whereas Roswell Park Memorial Institute 1640 (RPMI‐1640) medium was used for *Candida* strains. The extract was serially diluted with an automated pipette to obtain final concentrations between 25 mg/mL and 0.024 mg/mL. Tigecycline and voriconazole served as reference agents for bacteria and yeasts, respectively, and were tested over a concentration range of 512–0.5 μg/mL. Each assay was performed in triplicate on independent microplates. After incubation at 37°C for 24 h for bacteria and at 27°C for 48 h for yeasts, 50 μL of 2,3,5‐triphenyltetrazolium chloride (TTC, 2 mg/mL) was added to each well to assess microbial viability. Following an additional 30 min of incubation, MIC values were recorded according to the observed color change. RPMI‐1640 medium was included as the negative control.

### 2.6 Spectrophotometric Determination of Total Phenolic and Flavonoid Contents

The phenolic and flavonoid richness of the *S. germanica* extract was evaluated by spectrophotometric assays following the procedures reported by Uysal et al. (2016). Gallic acid was used to generate the calibration curve for total phenolic content (TPC), while rutin served as the reference compound for total flavonoid content (TFC). The results were calculated from the corresponding standard curves and expressed as mg gallic acid equivalents per g dry extract (mg GAE/g) and mg rutin equivalents per g dry extract (mg RE/g), respectively.

### 2.7 LC–MS/MS‐Based Quantification of Major Metabolites

#### 2.7.1 Preparation of Standards and Plant Extract Solutions

Primary stock solutions of the reference compounds were prepared at 1.0 mg/mL. Based on their solubility, hesperidin, kaempferol‐3‐glucoside, apigenin‐7‐glucoside, vitexin, chrysin, ellagic acid, and chlorogenic acid were dissolved in dimethyl sulfoxide (DMSO), whereas the remaining standards were prepared in high‐performance liquid chromatography (HPLC)‐grade ethanol. Working calibration solutions were obtained by serial dilution of the stock solutions with ethanol and stored at −20°C until analysis. For sample preparation, 50 mg of dry extract was accurately weighed into centrifuge tubes and extracted with 5 mL of HPLC‐grade ethanol. The mixture was homogenized by vortex mixing for 1 min and subsequently shaken on a mechanical shaker for 10 min to improve solvent penetration. Ultrasonic‐assisted extraction was then performed for 15 min. After extraction, the samples were centrifuged at 5000 rpm for 10 min at 20°C to separate solid residues. The supernatant was carefully collected and passed through a 0.45 μm polytetrafluoroethylene (PTFE) syringe filter. When required, the filtered sample solutions were further diluted with HPLC‐grade ethanol to ensure that analyte responses remained within the corresponding calibration ranges. The applied dilution factors were considered in the final quantitative calculations. The final solutions were transferred into autosampler vials prior to LC–MS/MS analysis. The solvent used for each reference standard, calibration concentration range, regression equation, and related quantitative information are summarized in Table S1.

#### 2.7.2 LC–MS/MS Instrumental Conditions and Method

Quantitative LC–MS/MS analyses were performed using a previously established method reported by İnan et al. (2026). The system consisted of an Agilent 6460 triple quadrupole mass spectrometer equipped with an electrospray ionization (ESI) source and coupled to an Agilent 1260 HPLC system (Agilent Technologies, Santa Clara, CA, USA). Chromatographic separation was achieved on an Inertsil ODS‐3 analytical column (4.6 × 250 mm, 5 μm; GL Sciences, Tokyo, Japan), and the column temperature was maintained at 40°C during the analysis. The mobile phase system consisted of solvent A (methanol containing 5 mM ammonium formate and 0.1% formic acid) and solvent B (ultrapure water supplemented with 5 mM ammonium formate and 0.1% formic acid). The flow rate was set at 0.5 mL/min and the injection volume was fixed at 5 μL. A gradient elution program with a total run time of 30 min was applied as follows: the mobile phase was held at 10% A from 0.0 to 3.0 min, increased linearly from 10% A to 90% A between 3.0 and 10.0 min, maintained at 90% A from 10.0 to 22.0 min, returned to 10% A at 22.1 min, and then kept at 10% A until 30.0 min for column re‐equilibration.

#### 2.7.3 Mass Spectrometry, Software, and Source Parameters

Analyte detection was performed using an electrospray ionization (ESI) source operated in both positive and negative ion modes. Quantification was based on multiple reaction monitoring (MRM) transitions selected for each target compound. Data acquisition, compound confirmation, calibration curve generation, and quantitative processing were performed using the Agilent MassHunter Workstation software. The ESI source operating conditions were optimized prior to analysis and set as follows: drying gas temperature, 300°C; drying gas flow rate, 8 L/min; sheath gas temperature, 300°C; sheath gas flow rate, 12 L/min; and nebulizer pressure, 40 psi. The capillary voltage was set to 4500 V, while the nozzle voltage was maintained at 2000 V. Calibration curves and representative LC–MS/MS chromatograms of the quantified compounds are provided in Figures S1–S5.

### 2.8 Evaluation of Antioxidant Activity

The antioxidant capacity of the extract was evaluated using complementary in vitro assays described by Zengin and Aktumsek (2014). Radical‐scavenging activity was assessed by 2,2‐diphenyl‐1‐picrylhydrazyl (DPPH) and 2,2′‐azino‐bis (3‐ethylbenzothiazoline‐6‐sulfonic acid) (ABTS) assays, whereas reducing power was determined using ferric reducing antioxidant power (FRAP) and cupric reducing antioxidant capacity (CUPRAC) assays. The phosphomolybdenum (PBD) assay was used to estimate total antioxidant capacity, and metal‐chelating activity (MCA) was evaluated as an additional mechanism‐related antioxidant parameter. Results were expressed as Trolox equivalents (TE) for radical‐scavenging, reducing power, and phosphomolybdenum assays, and as ethylenediaminetetraacetic acid equivalents (EDTAE) for metal‐chelating activity.

### 2.9 Assessment of Enzyme‐Inhibitory Activities

The enzyme‐inhibitory potential of the extract was evaluated according to the procedures reported by Çakmak et al. (2017). Cholinesterase inhibition was assessed against acetylcholinesterase (AChE) and butyrylcholinesterase (BChE), and the results were expressed as mg galanthamine equivalents per g extract (mg GALAE/g). Inhibitory activity against the carbohydrate‐hydrolyzing enzymes α‐amylase and α‐glucosidase was calculated as mmol acarbose equivalents per g extract (mmol ACAE/g). Tyrosinase inhibition was determined separately and reported as mg kojic acid equivalents per g extract (mg KAE/g).

### 2.10 Statistics

All measurements were performed in triplicate as analytical replicates from a single extract preparation. Results are expressed as mean ± standard deviation. Statistical evaluation of the data was performed using one‐way analysis of variance (ANOVA) followed by Tukey's multiple comparison post hoc test to determine significant differences among the extract groups. Data analysis was conducted using the GraphPad Prism software (Version 9.2), and statistical significance was accepted at a threshold of *p* < 0.05.

## 3 Results

### 3.1 Assessment of the Content of Phenolic Profile

The total phenolic content (TPC) and total flavonoid content (TFC) of *S. germanica* were quantitatively determined to evaluate its polyphenolic richness and to position the species within the broader phytochemical framework of the genus *Stachys*. The TPC of the extract was measured as 59.80 ± 0.84 mg gallic acid equivalents (GAE) per g extract, while the TFC was determined as 20.50 ± 0.24 mg rutin equivalents (RE) per g extract. These values indicate that *S. germanica* represents a phenolic‐rich taxon, with flavonoids accounting for a moderate proportion of the total phenolic pool.

### 3.2 Antioxidant Activity Assays

The antioxidant capacity of the extract was evaluated using multiple complementary in vitro assays based on different reaction mechanisms (Tables 1 and 2). In radical‐scavenging assays, the extract demonstrated DPPH activity of 333.96 ± 1.61 mg TE/g extract, whereas ABTS radical‐scavenging activity was higher, at 387.12 ± 3.03 mg TE/g extract. The reducing power assays further supported antioxidant capacity. The CUPRAC value was 445.26 ± 6.11 mg TE/g extract, representing the highest antioxidant capacity, followed by the FRAP value of 360.89 ± 5.19 mg TE/g extract. In contrast, metal‐chelating activity (MCA) was comparatively lower, measured at 16.32 ± 1.43 mg EDTAE/g extract. The total antioxidant capacity assessed by the phosphomolybdenum assay (PBD) was 1.47 ± 0.03 mmol TE/g extract. Overall, the extract exhibited consistent antioxidant responses in radical‐scavenging and reducing assays, with CUPRAC showing the highest magnitude among the evaluated parameters.

**TABLE 1:** Total phenolic and flavonoid contents and radical‐scavenging effects of *S. germanica* extract.

Species	TPC (mg GAE/g)	TFC (mg RE/g)	DPPH (mg TE/g)	ABTS (mg TE/g)
*S. germanica*	59.80 ± 0.84	20.50 ± 0.24	333.96 ± 1.61	387.12 ± 3.03

*Note:* Values are expressed as mean ± standard deviation of triplicate analytical measurements from a single extract preparation.

Abbreviations: GAE, gallic acid equivalents; RE, rutin equivalents; TE, trolox equivalents.

**TABLE 2:** Reducing, metal chelating, and total antioxidant (by phosphomolybdenum) effects of *S. germanica* extract.

Species	CUPRAC (mg TE/g)	FRAP (mg TE/g)	MCA (mg EDTAE/g)	PBD (mmol TE/g)
*S. germanica*	445.26 ± 6.11	360.89 ± 5.19	16.32 ± 1.43	1.47 ± 0.03

*Note:* Values are expressed as mean ± standard deviation of triplicate analytical measurements from a single extract preparation.

Abbreviations: EDTAE, ethylenediaminetetraacetic acid equivalents; TE, trolox equivalents.

### 3.3 Enzyme Inhibitory Activities

The inhibitory effects of the extract were assessed against enzymes associated with neurodegenerative, metabolic, and dermatological targets (Table 3). The extract demonstrated measurable acetylcholinesterase (AChE) inhibition of 2.73 ± 0.03 mg GALAE/g extract, while butyrylcholinesterase (BChE) inhibition was slightly lower at 2.23 ± 0.09 mg GALAE/g extract. Tyrosinase inhibition was comparatively more pronounced, reaching 62.23 ± 0.06 mg KAE/g extract. Regarding carbohydrate‐hydrolyzing enzymes, α‐amylase inhibition was recorded as 0.16 ± 0.01 mmol ACAE/g extract, whereas α‐glucosidase inhibition was substantially higher at 1.05 ± 0.24 mmol ACAE/g extract. Among the evaluated enzymes, tyrosinase and α‐glucosidase inhibition represented the most prominent activities, while α‐amylase inhibition was comparatively limited.

**TABLE 3:** Enzyme inhibitory effects of *S. germanica* extract.

Species	AChE (mg GALAE/g)	BChE (mg GALAE/g)	Tyrosinase (mg KAE/g)	Amylase (mmol ACAE/g)	Glucosidase (mmol ACAE/g)
*S. germanica*	2.73 ± 0.03	2.23 ± 0.09	62.23 ± 0.06	0.16 ± 0.01	1.05 ± 0.24

*Note:* Values are expressed as mean ± standard deviation of triplicate analytical measurements from a single extract preparation.

Abbreviations: ACAE, acarbose equivalent; GALAE, galantamine equivalent; KAE, kojic acid equivalent.

### 3.4 Anti‐Microbial Activity

The antimicrobial activity of the 80% ethanolic extract of *S. germanica* was evaluated against a panel of Gram‐positive bacteria, Gram‐negative bacteria, and yeast strains using the broth microdilution method (Table 4). For Gram‐positive bacteria, minimum inhibitory concentration (MIC) values ranged from 1562 to 25,000 μg/mL. The lowest MIC value within this group was observed against *C. striatum* (clinical isolate) at 1562 μg/mL, followed by *S. aureus* ATCC 29213 and clinical *S. aureus* strains (3125 μg/mL). Higher MIC values were recorded for *S. epidermidis* ATCC 12228 (6250 μg/mL) and *B. subtilis* ATCC 6633 (25,000 μg/mL), indicating reduced susceptibility among certain reference strains. In the Gram‐negative group, MIC values showed a broader distribution, ranging from 97 μg/mL to 25,000 μg/mL. The lowest MIC value was observed against *A. baumannii* ATCC 19606, with an MIC value of 97 μg/mL, followed by *P. aeruginosa* ATCC 27853 (390 μg/mL) and *E. coli* ATCC 25922 (781 μg/mL). Clinical isolates of *E. coli* , *P. aeruginosa* , and *A. baumannii* exhibited higher MIC values (3125–25,000 μg/mL), indicating reduced susceptibility compared with certain ATCC strains. For yeast strains, MIC values ranged between 390 μg/mL and 6250 μg/mL. The lowest MIC was observed against *C. albicans* ATCC 10231 (390 μg/mL), followed by *C. parapsilosis* ATCC 22019 (781 μg/mL). Higher MIC values were recorded for *C. tropicalis* ATCC 750 (6250 μg/mL) and clinical *C. albicans* isolates (1562 μg/mL). According to the applied classification criteria (MIC < 100 μg/mL, significant; 100–625 μg/mL: moderate; > 625 μg/mL: weak), the extract demonstrated significant activity only against *A. baumannii* ATCC 19606, moderate activity against selected Gram‐negative and yeast strains, and predominantly weak activity against most Gram‐positive bacteria and clinical isolates.

**TABLE 4:** Minimum inhibitory concentrations (MIC) of *S. germanica* extract.

Antimicrobial Activity Results		
Microorganisms	Stachys germanica (μg/mL)	Tigecycline‐ Voriconazole (μg/mL)
Gram‐positive bacteria		
*Staphylococcus aureus* ATCC 29213	3125	0.5
*Staphylococcus epidermidis* ATCC 12228	6250	1
*Bacillus subtilis* ATCC 6633	25,000	1
*Corynebacterium striatum* (Clinic)	1562	2
*Staphylococcus aureus* (Clinic)	3125	16
*Staphylococcus epidermidis* (Clinic)	3125	2
Gram‐negative bacteria		
*Escherichia coli* ATCC 25922	781	0.5
*Salmonella enterica* ATCC 13076	781	2
*Acinetobacter baumannii ATCC 19606*	97	2
*Pseudomonas aeruginosa* ATCC 27853	390	32
*Escherichia coli* (Clinic)	3125	16
*Pseudomonas aeruginosa* (Clinic)	12,500	16
*Acinetobacter baumannii* (Clinic)	25,000	2
Yeast		
*Candida albicans* ATCC 10231	390	4
*Candida tropicalis* ATCC 750	6250	16
*Candida parapsilosis* ATCC 22019	781	4
*Candida albicans* (Clinic)	1562	≥ 256

*Note:* Significant activity: MIC < 100 μg/mL; moderate activity: 100 < MIC ≤ 625 μg/mL; weak activity: MIC > 625 μg/mL. Activity classification was based on previously reported MIC interpretation criteria (Eloff 2004; Kuete et al. 2011; Awouafack et al. 2013).

### 3.5 Quantification of Major Metabolites by LC–MS/MS

The quantitative LC–MS/MS analysis of the 80% ethanolic extract of *S. germanica* revealed a chemically diverse metabolite profile composed predominantly of organic acids, phenolic acids, flavonoids, and procyanidins (Table 5). Among the detected compounds, malic acid was the most abundant metabolite (729 ± 13.12 μg/g), dominating the overall phytochemical profile. Within the phenolic acid group, chlorogenic acid was detected at a notably high concentration (1024 ± 20.54 μg/g), representing the predominant phenolic constituent of the extract. This was followed by syringic acid (66 ± 1.42 μg/g), protocatechuic acid (51 ± 1.23 μg/g), and ellagic acid (79 ± 1.74 μg/g), indicating the significant presence of hydroxybenzoic and hydroxycinnamic acid derivatives. Flavonoid profiling demonstrated that naringin was the most abundant flavonoid glycoside (196.10 ± 4.65 μg/g), while luteolin (82.46 ± 1.88 μg/g) and kaempferol (70.99 ± 1.71 μg/g) were the major aglycone forms. Moderate concentrations of apigenin (39.73 ± 0.77 μg/g) and diosgenin (28 ± 0.68 μg/g) were also observed. Glycosylated flavonoids such as apigenin‐7‐glucoside (119.72 ± 3.45 μg/g) and kaempferol‐3‐glucoside (23.42 ± 0.62 μg/g) further contributed to the flavonoid pool. Compounds such as caffeic acid, trans‐ferulic acid, and trans‐p‐coumaric acid were detected at lower concentrations. Procyanidins and flavan‐3‐ol derivatives were present at relatively low levels, with procyanidin B1 (2 ± 0.04 μg/g) being the most abundant compound within this subgroup, followed by procyanidin B2 and procyanidin C1. Other minor constituents, including catechin hydrate, chrysin, and vitexin, were detected at trace levels. Overall, the LC–MS/MS results demonstrate that the metabolite profile of *S. germanica* is characterized by the predominance of chlorogenic acid and malic acid, supported by a structurally diverse flavonoid composition. This chemically heterogeneous profile suggests a synergistic contribution of multiple phenolic subclasses to the biological activities observed in the present study.

**TABLE 5:** Quantification of major metabolites and MRM transition parameters of *S. germanica* extract by LC–MS/MS.

Compounds	*S. germanica* extract	Precursor ion (m/z)	Product ions (m/z)
4‐hydroxybenzoic acid	5 ± 0.17	137.0 [M − H]^−^	93.0; 65.0
Syringic acid	66 ± 1.42	197.0 [M − H]^−^	123.0; 95.0
*Trans*‐ferulic acid	12 ± 0.28	193.0 [M − H]^−^	134.0; 178.0
Ellagic acid	79 ± 1.74	301.0 [M − H]^−^	145.0; 117.0
Chlorogenic acid	1024 ± 20.54	355.0 [M + H]^+^	163.0; 145.0
Cinnamic acid	22 ± 0.44	149.0 [M + H]^+^	131.0; 103.0
*Trans*‐p‐coumaric acid	10 ± 0.19	163.0 [M − H]^−^	119.0; 93.0
Caffeic acid	17 ± 0.24	179.0 [M − H]^−^	135.0; 89.0
Gallic acid	5 ± 0.11	169.0 [M − H]^−^	125.0; 79.0
Protocatechuic acid	51 ± 1.23	153.0 [M − H]^−^	109.0; 65.0
Procyanidin A1	0.35 ± 0.01	577.0 [M + H]^+^	287.0; 425.0
Procyanidin A2	0.32 ± 0.01	577.0 [M + H]^+^	287.0; 425.0
Procyanidin B1	2 ± 0.04	579.1 [M + H]^+^	127.0; 409.0
Procyanidin B2	0.9 ± 0.01	579.1 [M + H]^+^	127.0; 409.0
Procyanidin C1	0.66 ± 0.01	867.1 [M + H]^+^	579.1; 291.0
Catechin hydrate	0.29 ± 0.01	291.0 [M + H]^+^	139.0; 123.0
Malic acid	729 ± 13.12	133.0 [M − H]^−^	115.0; 71.0
Diosgenin	28 ± 0.68	415.2 [M + H]^+^	271.2; 253.2
Apigenin	39.73 ± 0.77	269.0 [M − H]^−^	117.1; 149.1
Luteolin	82.46 ± 1.88	285.0 [M − H]^−^	133.1; 151.0
Taxifolin	0.75 ± 0.02	303.0 [M − H]^−^	285.0; 125.0
Naringenin	6.06 ± 0.15	271.0 [M − H]^−^	119.1; 151.0
Kaempferol	70.99 ± 1.71	285.0 [M − H]^−^	285.0; 117.1
Quercetin	9.84 ± 0.24	301.0 [M − H]^−^	151.0; 178.9
Vitexin	0.03 ± 0.001	431.0 [M − H]^−^	311.0; 283.0
Chrysin	0.23 ± 0.01	253.0 [M − H]^−^	63.2; 143.1
Apigenin 7‐glucoside	119.72 ± 3.45	431.0 [M − H]^−^	268.0; 238.9
Kaempferol 3‐glucoside	23.42 ± 0.62	447.0 [M − H]^−^	284.0; 255.1
Naringin	196.10 ± 4.65	579.0 [M − H]^−^	271.0; 151.0
Hesperidin	0.79 ± 0.21	609.0 [M − H]^−^	301.0; 163.9
Rutin	6 ± 0.16	609.0 [M − H]^−^	299.9; 270.9

*Note:* Ionization mode is indicated as [M − H]^−^ or [M + H]^+^. Product ions are presented as the monitored MRM transitions used for compound confirmation and quantification. Values are expressed as mean ± standard deviation (μg/g dry extract).

## 4 Discussion

This study provides a comprehensive chemical and biological characterization of *Stachys germanica* , integrating phenolic quantification, antioxidant assays, enzyme inhibition screening, antimicrobial testing, and LC–MS/MS profiling into a coherent framework. Within the genus *Stachys*, secondary metabolite diversity—particularly flavonoids, phenolic acids, phenylethanoid glycosides, and iridoid derivatives—has been consistently reported as the major driver of pharmacological activity (Benedec et al. 2023; Háznagy‐Radnai et al. 2021). The phenolic‐rich composition and multifunctional activity profile observed in the present extract align with this chemotaxonomic background while simultaneously demonstrating species‐specific differences. The total phenolic and flavonoid contents determined here are consistent with values reported for Mediterranean and Balkan *Stachys* taxa, where hydroalcoholic extraction frequently enhances recovery of moderately polar flavonoids and phenolic acids (Bulut et al. 2025). Comparative chemical profiling studies have shown that flavones, flavonols, and their glycosides often dominate chromatographic fingerprints, although phenolic acids substantially contribute to total phenolic measurements (Tomou et al. 2020). Accordingly, the TPC–TFC distribution observed in *S. germanica* reflects a chemically heterogeneous matrix rather than the dominance of a single subclass of polyphenols. Similar phenolic distribution patterns, characterized by markedly higher TPC values relative to TFC, have been consistently documented for numerous *Stachys* species, including *S. chasmosericea*, *S. arvensis* , *S. brachyclada*, *S. annua* , *S. inflata* , and *S. cretica* subspecies (Sarikurkcu et al. 2021; Solmaz et al. 2025; Bulut et al. 2025). These findings collectively indicate that the polyphenolic profiles of *Stachys* taxa are characterized by the concurrent presence of phenolic acids, phenylethanoid glycosides, and flavone derivatives, with the relative predominance of these subclasses differing among species rather than being confined to a single dominant compound group. Previous comparative LC–HRMS studies on *Stachys* taxa have demonstrated substantial qualitative and quantitative variation in key constituents, including acteoside, caffeoylquinic acids, and flavone 7‐O‐allosylglucosides, across taxa, underscoring the chemical heterogeneity of the genus (Karioti et al. 2022). Overall, the quantitative assessment of total phenolic and flavonoid contents confirms that *S. germanica* exhibits a polyphenolic profile consistent with the broader chemotaxonomic framework of the genus *Stachys*. The predominance of total phenolics relative to flavonoids reflects a structurally diverse matrix in which multiple phenolic subclasses collectively contribute to the phytochemical architecture. A comparable quantitative distribution has been reported in other phenolic‐rich *Stachys* species evaluated by LC‐based profiling and spectrophotometric assays (Sarikurkcu et al. 2016).

The rich polyphenolic matrix of *S. germanica* is reflected in the pronounced antioxidant capacity observed in radical‐scavenging and reducing‐power assays. In *Stachys* species, antioxidant responses have consistently been associated with LC‐determined phenolic profiles, particularly caffeoylquinic acids, verbascoside derivatives, and flavone glycosides, which contribute to redox‐active behavior in ABTS, DPPH, and reducing power assays (Benabderrahim et al. 2021). In the present study, *S. germanica* exhibited measurable radical‐scavenging activity in both the DPPH and ABTS assays, indicating its capacity to neutralize stable free radicals via hydrogen atom or electron‐donation mechanisms. The ABTS scavenging activity was slightly higher than the DPPH response, a pattern frequently observed in *Stachys* species and often attributed to the broader reactivity of the ABTS radical toward both hydrophilic and lipophilic antioxidant compounds (Solmaz et al. 2025). Similar trends have been reported for *S. chasmosericea*, *S. arvensis* , and *S. cretica* subspecies, where ABTS values generally exceeded those obtained with DPPH, despite comparable total phenolic levels (Sarikurkcu et al. 2021; Bulut et al. 2025). The reducing power of *S. germanica* , as determined by CUPRAC and FRAP assays, indicates that its antioxidant activity is predominantly mediated by electron‐transfer–based mechanisms. The comparatively higher CUPRAC values relative to FRAP suggest a broader responsiveness of the extract to copper(II)‐reducing systems, which are known to detect both flavonoid and phenolic acid derivatives under near‐neutral conditions. Similar assay‐dependent differences between CUPRAC and FRAP responses have been documented in LC‐characterized *Stachys* extracts, highlighting the methodological influence on perceived reducing capacity (Bahadori et al. 2019). In addition to radical‐scavenging and reducing power activities, *S. germanica* displayed a measurable ferrous ion chelating capacity (16.32 ± 1.43 mg EDTAE/g extract), indicating that part of its antioxidant action may also involve limiting metal‐catalyzed oxidative processes rather than relying solely on electron‐transfer reactions. Notably, comparable chelation‐oriented responses have been documented in the genus under similar assay conditions; for example, solvent‐dependent chelating effects were reported for *Stachys* extracts, in which the chelating contribution was highlighted as a complementary antioxidant mechanism alongside reducing power and radical‐scavenging (Sarikurkcu et al. 2016). In a separate matrix (decoction model), hedgenettle teas (*S. byzantina, S. inflata*, and *S. lavandulifolia*) also exhibited quantifiable metal chelating activity (reported as mg EDTAE/g decoction), supporting that chelation can consistently emerge as a detectable—yet mechanism‐specific—component of the antioxidant profile across *Stachys* preparations (Bahadori et al. 2019). The total antioxidant capacity, measured by the phosphomolybdenum assay, further supported the redox potential of *S. germanica* (1.47 ± 0.03 mmol TE/g extract), reflecting the extract's integrated reducing ability rather than a single reaction pathway. Although the LC–MS/MS data support the contribution of quantified phenolic acids and flavonoids to the observed antioxidant and metal‐chelating effects, the overall in vitro activity may also be partially associated with other non‐flavonoid phenolics and unquantified metabolites, such as phenylethanoid glycosides, iridoids, and terpenoid constituents, which are commonly reported in *Stachys* species (Napolitano et al. 2022). The antioxidant performance of *S. germanica* can be rationally interpreted in relation to its total phenolic and flavonoid contents. The relatively high TPC value (59.80 ± 0.84 mg GAE/g extract) indicates a substantial pool of redox‐active compounds capable of contributing to radical‐scavenging and reducing power, while the moderate TFC (20.50 ± 0.24 mg RE/g extract) suggests that flavonoids represent only part of this antioxidant system. Comparative analysis with previously published data places *S. germanica* within the moderate‐to‐strong antioxidant category among *Stachys* species. Extracts of *S. chasmosericea* and selected *S. cretica* subspecies have been reported to exhibit higher antioxidant responses, particularly when very high phenolic loads were present, whereas other species such as *S. annua* , *S. inflata* , and *S. arvensis* displayed antioxidant capacities comparable to those observed in the present study (Nikolova 2011; Sarikurkcu et al. 2021; Bulut et al. 2025). These differences further emphasize the influence of species‐specific phytochemical composition, environmental factors, and extraction methodology on antioxidant outcomes. Overall, the results of the antioxidant assays demonstrate that *Stachys germanica* possesses a well‐balanced antioxidant profile characterized by effective radical‐scavenging and reducing power, supported by its substantial total phenolic content. The observed antioxidant activities are in good agreement with those reported for other medicinally relevant *Stachys* taxa and provide a solid biochemical basis for the traditional use of this genus in conditions associated with oxidative stress.

The enzyme inhibitory potential of *S. germanica* was evaluated against a panel of key enzymes associated with metabolic disorders, neurodegenerative diseases, and dermatological conditions, including acetylcholinesterase (AChE), butyrylcholinesterase (BChE), α‐amylase, α‐glucosidase, and tyrosinase. In the present study, *S. germanica* exhibited measurable inhibitory activity against both cholinesterases, with a slightly stronger effect on AChE than on BChE. Similar cholinesterase inhibitory profiles have been reported for several *Stachys* species, including *S. lavandulifolia*, *S. byzantina* , and *S. cretica* subspecies, supporting the view that members of the genus possess compounds capable of modulating cholinergic enzymes (Nikolova 2011; Mohammadi and Kharazian 2022; Bulut et al. 2025). Although the inhibitory potency of *S. germanica* is lower than that of standard drugs, its activity is consistent with values reported for other medicinally relevant *Stachys* taxa and may be of interest in the context of mild or complementary neuroprotective strategies. The inhibitory effects observed against α‐glucosidase were more pronounced than those recorded for α‐amylase, indicating a selective inhibitory profile toward carbohydrate‐digesting enzymes. This pattern is considered advantageous, as strong α‐glucosidase inhibition combined with moderate or weak α‐amylase inhibition may help regulate postprandial hyperglycemia while minimizing gastrointestinal side effects (Sarikurkcu et al. 2021; Bulut et al. 2025). Similar enzyme selectivity has been documented for *S. chasmosericea*, *S. arvensis* , and *S. brachyclada*, where α‐glucosidase inhibition was consistently stronger than α‐amylase inhibition (Sarikurkcu et al. 2021; Solmaz et al. 2025). Tyrosinase inhibition by *S. germanica* was observed at a moderate level, suggesting potential relevance to applications for hyperpigmentation and oxidative skin damage. Comparable tyrosinase‐inhibitory activities have been reported for various *Stachys* species, including Balkan and Anatolian taxa, and are generally attributed to polyphenolic constituents with redox‐modulating properties (Háznagy‐Radnai et al. 2005; Sarikurkcu et al. 2021). The enzyme inhibitory activities observed in the present study can be mechanistically explained by the combined action of phenolic acids and flavonoids, which target enzymes through multiple complementary pathways. Phenolic acids such as chlorogenic and caffeic acid primarily exert their effects through mixed‐type inhibition, involving simultaneous interaction with the catalytic site and allosteric regions of the enzyme, leading to conformational alterations that reduce enzymatic activity (Shi et al. 2025). In parallel, flavonoids inhibit enzymes through direct binding near or within the active site, forming stable enzyme–inhibitor complexes that block substrate access and interfere with catalytic processes. These interactions are largely governed by hydrophobic forces and hydrogen bonding and are influenced by structural features such as hydroxyl group distribution and conjugated systems (Martinez‐Gonzalez et al. 2019). Moreover, both phenolic acids and flavonoids may exert dual inhibitory mechanisms by interacting not only with enzymes but also with substrates, particularly with carbohydrate‐digesting enzymes such as α‐amylase and α‐glucosidase, where binding to starch or intermediate substrates can further reduce enzymatic efficiency (Aleixandre et al. 2022). Taken together, the enzyme‐inhibitory activity of the extract can be attributed to a multi‐target inhibitory system, in which phenolic acids primarily induce conformational and redox‐related effects, while flavonoids provide strong active‐site interactions, resulting in an overall enhancement of inhibitory potential through complementary mechanisms.

The LC–MS/MS analysis revealed that the extract of *Stachys germanica* is characterized by a phenolic‐rich composition, with chlorogenic acid identified as the predominant phenolic acid, accompanied by flavonoids such as luteolin, kaempferol, and naringin. This profile indicates that both phenolic acids and flavonoids contribute to the extract's overall chemical structure. This predominance is consistent with previous phytochemical investigations of *Stachys germanica* , where chlorogenic acid was identified as one of the major metabolites across different extracts and chromatographic profiles (Sarikurkcu et al. 2021). In addition to phenolic acids, flavonoids represent another important component of the extract. Previous metabolomic and phytochemical studies have demonstrated that *Stachys* species are particularly rich in flavonoids, especially flavones and flavonols, which are widely distributed across the genus (Mohammadi and Kharazian 2022). The presence of luteolin and kaempferol in the current study is therefore consistent with the general phytochemical profile reported for *Stachys*. Overall, the LC–MS/MS results confirm that *S. germanica* exhibits a typical *Stachys*‐type phenolic profile, dominated by chlorogenic acid and supported by structurally diverse flavonoids. This composition is consistent with previously reported data for the genus and provides a chemical basis for the biological activities observed in the present study. The tyrosinase inhibition observed for *S. germanica* therefore aligns well with the enzyme inhibitory spectrum previously described for the genus. The enzyme‐inhibitory behavior of *S. germanica* can be partially attributed to its total phenolic and flavonoid contents. The substantial total phenolic content (59.80 ± 0.84 mg GAE/g extract) suggests a sufficient concentration of bioactive compounds capable of interacting with enzyme active sites or modulating enzyme conformation, whereas the moderate flavonoid content (20.50 ± 0.24 mg RE/g extract) indicates that non‐flavonoid phenolics may play a prominent role in the observed inhibitory effects. Comparative evaluation with published data reveals that the enzyme‐inhibitory profile of *S. germanica* is broadly comparable to that reported for other *Stachys* taxa, rather than representing an outlier within the genus. Extracts of *S. chasmosericea* and *S. cretica* subspecies have shown stronger inhibition toward enzymes related to melanogenesis, carbohydrate digestion, and cholinergic function, particularly tyrosinase, α‐glucosidase, and cholinesterases, when higher phenolic loads were present, whereas *S. annua* , *S. inflata* , and *S. arvensis* exhibited enzyme inhibitory activities within a similar range to those observed in the present study (Nikolova 2011; Sarikurkcu et al. 2021; Bulut et al. 2025). These differences further emphasize the influence of species‐specific phytochemical composition and extraction parameters on enzyme inhibition outcomes. Overall, the enzyme inhibition results demonstrate that *Stachys germanica* exhibits a multi‐target inhibitory profile, affecting enzymes related to neurodegeneration, carbohydrate metabolism, and melanogenesis. While the observed activities are moderate, they are consistent with the species' phenolic‐rich nature and align well with enzyme‐inhibitory patterns previously reported for the genus *Stachys*. These findings support the potential of *S. germanica* as a source of natural compounds with complementary enzyme‐modulating properties and provide a mechanistic basis for further pharmacological investigations.

The antimicrobial activity of *Stachys germanica* may be partly associated with its phenolic‐rich composition, particularly the presence of phenolic acids, including chlorogenic acid and related compounds, as well as flavonoids. In the present study, however, significant activity was observed only against *A. baumannii* ATCC 19606, while the extract showed mostly weak or moderate activity against the remaining tested microorganisms. Therefore, the antimicrobial findings should be interpreted as a limited but selective inhibitory effect rather than broad‐spectrum antimicrobial activity. The selective response observed against *A. baumannii* ATCC 19606 may be related, at least in part, to the reported ability of phenolic acids to affect bacterial redox balance. Phenolic acids containing catechol structures have been shown to act as pro‐oxidants, promoting the generation of reactive oxygen species (ROS) such as superoxide anions. This leads to oxidative stress, disruption of cellular redox homeostasis, and damage to essential biomolecules including DNA, proteins, and membrane lipids. In *A. baumannii* , this mechanism is particularly relevant, as increased ROS production has been directly associated with enhanced bacterial killing and metabolic disruption (Ajiboye et al. 2018). In addition to phenolic acids, flavonoids present in the extract may contribute to antimicrobial activity through antibiofilm and antivirulence effects. Previous studies have shown that flavonoids can inhibit biofilm formation, surface motility, and virulence‐related regulatory systems in *A. baumannii* , thereby reducing its pathogenicity without necessarily exerting strong bactericidal effects. This suggests that flavonoids may complement the direct antimicrobial effects of phenolic acids by targeting bacterial survival strategies such as biofilm formation. Taken together, these observations suggest that the selective activity of *S. germanica* against *A. baumannii* ATCC 19606 may involve complementary effects of phenolic acids and flavonoids. Nevertheless, considering that significant activity was observed against only one of the 17 tested strains, these mechanistic interpretations should be considered preliminary. Further studies using bioactivity‐guided fractionation, isolated compounds, and additional clinical isolates are required to confirm the active constituents and clarify their mechanisms of action.

## 5 Conclusion

This study provides an integrated chemical and biological characterization of *Stachys germanica* by combining quantitative phytochemical profiling with complementary antioxidant, enzyme inhibition, and antimicrobial assays. The findings demonstrate that this underexplored species has a phenolic‐rich and chemically diverse profile associated with multifunctional biological effects. Rather than being attributable to a single dominant compound, the observed activities appear to reflect the cumulative contribution of several phenolic constituents and possibly other unquantified metabolites within a heterogeneous phytochemical matrix. In conclusion, these findings position *Stachys germanica* as a phenolic‐rich species exhibiting a balanced spectrum of antioxidant, enzyme‐modulating, and selective antibacterial activities. The results contribute to the expanding phytochemical knowledge of the genus *Stachys* and provide a foundation for future bioactivity‐guided fractionation, mechanistic investigations, and phytochemical standardization studies. Future work should also include the characterization of additional metabolite classes not covered by the current targeted analysis, as well as safety, bioaccessibility, and formulation‐related evaluations. In addition, assessment of geographical, seasonal, and ecological variability may help clarify the stability and reproducibility of the bioactive profile of *Stachys germanica* , thereby supporting its potential application in nutraceutical, functional food, or cosmeceutical formulations.

## Author Contributions

**Yiğit İnan:** investigation, methodology, conceptualization. **Gözde Gülin İnan:** writing – original draft, data curation, investigation, software, validation. **Esra Mertoğlu:** investigation. **Derya Doğanay:** investigation. **Durişehvar Özer Ünal:** writing – review and editing, supervision. **Neşet Neşetoğlu:** investigation, validation, software. **Selin Akyüz‐Çetin:** investigation, methodology, conceptualization. **İbrahim Daniş:** investigation, validation, software. **Gokhan Zengin:** writing – review and editing, supervision.

## Disclosure

Statement on the Use of Generative AI and AI‐Assisted Technologies: The authors declare that no generative artificial intelligence tools were used to create scientific content, generate data, perform analyses, interpret results, or draw conclusions in this manuscript. During manuscript preparation, standard non‐generative digital tools, such as spelling and grammar checkers available in Office 365 and Google Docs, as well as reference management software, were used only to support language accuracy, formatting, and citation organization. All text, data, interpretations, and conclusions were reviewed, verified, and approved by the authors, who take full responsibility for the final content of the manuscript.

## Conflicts of Interest

We declare that we have no known competing financial interests or personal relationships that could have appeared to influence the work reported in this paper. All authors of this manuscript have directly participated in the program execution and/or analysis of this study. The content of this manuscript has not been copyrighted or published before. The content of this manuscript is not currently considered for publication elsewhere.

## Supporting information


**Table S1:** Solvent, calibration range, and calibration equation of detected and quantified compounds analyzed by LC–MS/MS.
**Figure S1:** Calibration curves and representative LC–MS/MS chromatograms of phenolic acids: (A) gallic acid, (B) chlorogenic acid, (C) protocatechuic acid, (D) ellagic acid, (E) caffeic acid, (F) 4‐hydroxybenzoic acid, (G) syringic acid, (H) trans‐*p*‐coumaric acid, (I) trans‐ferulic acid, and (J) cinnamic acid.
**Figure S2:** Calibration curves and representative LC–MS/MS chromatograms of catechin and procyanidins: (A) catechin hydrate, (B) procyanidin A1, (C) procyanidin A2, (D) procyanidin B1, (E) procyanidin B2, and (F) procyanidin C1.
**Figure S3:** Calibration curves and representative LC–MS/MS chromatograms of flavonoid aglycones: (A) kaempferol, (B) apigenin, (C) chrysin, (D) luteolin, (E) naringenin, (F) quercetin, and (G) taxifolin.
**Figure S4:** Calibration curves and representative LC–MS/MS chromatograms of flavonoid glycosides: (A) vitexin, (B) rutin, (C) naringin, (D) kaempferol‐3‐glucoside, (E) hesperidin, and (F) apigenin‐7‐glucoside.
**Figure S5:** Calibration curves and representative LC–MS/MS chromatograms of (A) diosgenin and (B) malic acid.

## Data Availability

All data generated or analyzed during this study are included in this published article and its Supporting Information files. Raw LC–MS/MS chromatograms, peak integration data, calibration results, and quantitative measurements are provided as Supporting Information to ensure transparency and reproducibility.
