# Supplementary material for: Phytochemical Characterization and Bioactive Potential of Stachys germanica L.: LC–MS/MS Profiling and Multi‐Target Biological Activities
Source: Food Sci Nutr. 2026 Aug 7;14(8):e72238. doi: 10.1002/fsn3.72238 (PMC13451848; doi:10.1002/fsn3.72238)
Supplement: Supplementary file 1 [file FSN3-14-e72238-s001.docx]

**Supplementary Material**

**Phytochemical characterization and bioactive potential of *Stachys germanica* L.: LC–MS/MS profiling and multi-target biological activities**

**Supplementary LC–MS/MS Calibration Data, MRM Parameters, and Representative Chromatograms**

**Supplementary Table S1.** Solvent, calibration range, and calibration equation of detected and quantified compounds analyzed by LC–MS/MS.

| **Compound** | **Solvent used for stock solution** | **Calibration range (ng/mL)** | **Regression equation (y = ax + b)** | **R²** |
| --- | --- | --- | --- | --- |
| 4-Hydroxybenzoic acid | HPLC-grade ethanol | 10–5000 | y = 305.71x + 30579.67 | 0.9967 |
| Syringic acid | HPLC-grade ethanol | 10–5000 | y = 20.27x − 1094.03 | 0.9984 |
| *trans*-Ferulic acid | HPLC-grade ethanol | 10–5000 | y = 73.32x + 164.22 | 0.9999 |
| Ellagic acid | DMSO | 10–5000 | y = 5.27x − 587.55 | 0.9984 |
| Chlorogenic acid | DMSO | 10–5000 | y = 3684.99x + 868.75 | 0.9951 |
| Cinnamic acid | HPLC-grade ethanol | 10–5000 | y = 11.78x + 239.79 | 0.9983 |
| *trans*-p-Coumaric acid | HPLC-grade ethanol | 10–5000 | y = 402.47x + 34370.29 | 0.9982 |
| Caffeic acid | HPLC-grade ethanol | 10–5000 | y = 499.57x + 46157.33 | 0.9961 |
| Gallic acid | HPLC-grade ethanol | 10–5000 | y = 237.83x − 8900.08 | 0.9999 |
| Protocatechuic acid | HPLC-grade ethanol | 10–5000 | y = 380.74x + 9889.21 | 0.9978 |
| Procyanidin A1 | HPLC-grade ethanol | 10–5000 | y = 3098.23x + 32614.59 | 0.9968 |
| Procyanidin A2 | HPLC-grade ethanol | 10–5000 | y = 2977.10x + 16427.69 | 0.9969 |
| Procyanidin B1 | HPLC-grade ethanol | 10–5000 | y = 756.80x + 3552.45 | 0.9980 |
| Procyanidin B2 | HPLC-grade ethanol | 10–5000 | y = 1366.72x − 25882.00 | 0.9992 |
| Procyanidin C1 | HPLC-grade ethanol | 10–5000 | y = 454.72x − 10421.25 | 0.9974 |
| Catechin hydrate | HPLC-grade ethanol | 10–5000 | y = 1941.92x− 151571.38 | 0.9974 |
| Malic acid | HPLC-grade ethanol | 10–5000 | y = 123.40x − 16518.87 | 0.9974 |
| Diosgenin | HPLC-grade ethanol | 10–5000 | y = 531.77x − 77798.02 | 0.9983 |
| Apigenin | HPLC-grade ethanol | 10–5000 | y = 508.19x − 890.15 | 0.9996 |
| Luteolin | HPLC-grade ethanol | 10–5000 | y = 371.78x − 30.13 | 0.9994 |
| Taxifolin | HPLC-grade ethanol | 10–5000 | y = 288.19x + 1209.13 | 0.9993 |
| Naringenin | HPLC-grade ethanol | 10–5000 | y = 589.55x + 2339.23 | 0.9996 |
| Kaempferol | HPLC-grade ethanol | 10–5000 | y = 33.95x − 265.53 | 0.9999 |
| Quercetin | HPLC-grade ethanol | 10–5000 | y = 159.58x + 827.26 | 0.9993 |
| Vitexin | DMSO | 10–5000 | y = 321.35x + 615.69 | 0.9997 |
| Chrysin | DMSO | 10–5000 | y = 636.10x + 2773.09 | 0.9999 |
| Apigenin-7-glucoside | DMSO | 25–5000 | y = 399.41x + 8977.98 | 0.9969 |
| Kaempferol-3-glucoside | DMSO | 25–5000 | y = 66.32x + 1681.80 | 0.9977 |
| Naringin | HPLC-grade ethanol | 25–5000 | y = 28.51x − 363.28 | 0.9992 |
| Hesperidin | DMSO | 25–5000 | y = 46.60x − 608.35 | 0.9990 |
| Rutin | HPLC-grade ethanol | 25–5000 | y = 27.55x − 264.69 | 0.9992 |

***** DMSO: dimethyl sulfoxide. Calibration ranges are expressed as ng/mL. All reference standards were obtained from Sigma-Aldrich (St. Louis, MO, USA).

**A)**

**B)**

**C)**

**D)**

**E)**

**F)**

**G)**

**H)**

**I)**

**J)**

**Supplementary Figure S1.** Calibration curves and representative LC–MS/MS chromatograms of phenolic acids: (A) gallic acid, (B) chlorogenic acid, (C) protocatechuic acid, (D) ellagic acid, (E) caffeic acid, (F) 4-hydroxybenzoic acid, (G) syringic acid, (H) trans-*p*-coumaric acid, (I) trans-ferulic acid, and (J) cinnamic acid.

**A)**

**B)**

**C)**

**D)**

**E)**

**F)**

**Supplementary Figure S2.** Calibration curves and representative LC–MS/MS chromatograms of catechin and procyanidins: (A) catechin hydrate, (B) procyanidin A1, (C) procyanidin A2, (D) procyanidin B1, (E) procyanidin B2, and (F) procyanidin C1.

**A)**

**B)**

**C)**

**D)**

**E)**

**F)**

**G)**

**Supplementary Figure S3.** Calibration curves and representative LC–MS/MS chromatograms of flavonoid aglycones: (A) kaempferol, (B) apigenin, (C) chrysin, (D) luteolin, (E) naringenin, (F) quercetin, and (G) taxifolin.

**A)**

**B)**

**C)**

**D)**

**E)**

**F)**

**Supplementary Figure S4.** Calibration curves and representative LC–MS/MS chromatograms of flavonoid glycosides: (A) vitexin, (B) rutin, (C) naringin, (D) kaempferol-3-glucoside, (E) hesperidin, and (F) apigenin-7-glucoside.

**A)**

**B)**

**Supplementary Figure S5.** Calibration curves and representative LC–MS/MS chromatograms of (A) diosgenin and (B) malic acid.
